# [(*Z*)-*O*-Methyl *N*-(3-chloro­phen­yl)thio­carbamato-κ*S*](triphenyl­phosphine-κ*P*)gold(I)

**DOI:** 10.1107/S1600536810017319

**Published:** 2010-05-19

**Authors:** Primjira P. Tadbuppa, Edward R. T. Tiekink

**Affiliations:** a Department of Chemistry, National University of Singapore, Singapore 117543; b Department of Chemistry, University of Malaya, 50603 Kuala Lumpur, Malaysia

## Abstract

The Au atom in the title compound, [Au(C_8_H_7_ClNOS)(C_18_H_15_P)], exists within a slightly distorted linear geometry defined by an *S*,*P*-donor set [S—Au—P angle = 174.61 (4)°], with the distortion related to a short intra­molecular Au⋯O contact [2.988 (3) Å]. In the crystal structure, mol­ecules are arranged into supra­molecular chains along the *b* axis by C—H⋯π inter­actions.

## Related literature

For the structural systematics and luminescence properties of phosphinegold(I) carbonimidothio­ates, see: Ho *et al.* (2006 ▶); Ho & Tiekink (2007 ▶); Kuan *et al.* (2008 ▶). For the synthesis, see: Hall *et al.* (1993 ▶).
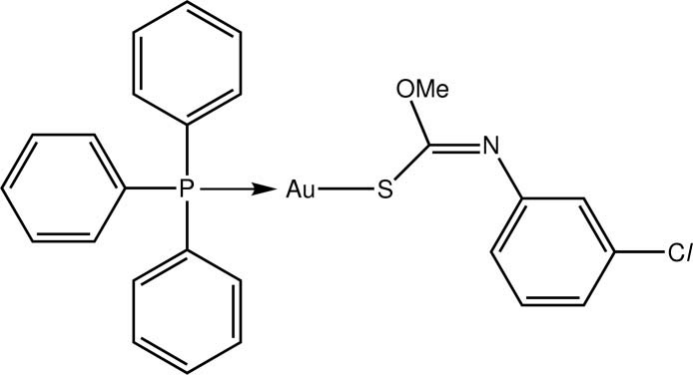


## Experimental

### 

#### Crystal data

[Au(C_8_H_7_ClNOS)(C_18_H_15_P)]
*M* *_r_* = 659.89
Triclinic, 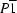

*a* = 10.4236 (8) Å
*b* = 10.6961 (8) Å
*c* = 12.7439 (9) Å
α = 72.724 (2)°
β = 66.105 (1)°
γ = 72.530 (2)°
*V* = 1213.83 (16) Å^3^
*Z* = 2
Mo *K*α radiation
μ = 6.34 mm^−1^
*T* = 223 K
0.19 × 0.08 × 0.05 mm

#### Data collection

Bruker SMART CCD diffractometer
Absorption correction: multi-scan (*SADABS*; Bruker, 2000 ▶) *T*_min_ = 0.515, *T*_max_ = 1
8630 measured reflections
5525 independent reflections
4787 reflections with *I* > 2σ(*I*)
*R*_int_ = 0.023

#### Refinement

*R*[*F* ^2^ > 2σ(*F* ^2^)] = 0.032
*wR*(*F* ^2^) = 0.089
*S* = 1.05
5525 reflections
290 parameters
H-atom parameters constrained
Δρ_max_ = 1.16 e Å^−3^
Δρ_min_ = −1.21 e Å^−3^

### 

Data collection: *SMART* (Bruker, 2000 ▶); cell refinement: *SAINT* (Bruker, 2000 ▶); data reduction: *SAINT*; program(s) used to solve structure: *PATTY* in *DIRDIF92* (Beurskens *et al.*, 1992 ▶); program(s) used to refine structure: *SHELXL97* (Sheldrick, 2008 ▶); molecular graphics: *ORTEP-3* (Farrugia, 1997 ▶) and *DIAMOND* (Brandenburg, 2006 ▶); software used to prepare material for publication: *publCIF* (Westrip, 2010 ▶).

## Supplementary Material

Crystal structure: contains datablocks global, I. DOI: 10.1107/S1600536810017319/lh5042sup1.cif

Structure factors: contains datablocks I. DOI: 10.1107/S1600536810017319/lh5042Isup2.hkl

Additional supplementary materials:  crystallographic information; 3D view; checkCIF report

## Figures and Tables

**Table 1:** Hydrogen-bond geometry (Å, °) *Cg*1 and *Cg*2 are the centroids of the C2–C7 and C9–C14 rings, respectively.

*D*—H⋯*A*	*D*—H	H⋯*A*	*D*⋯*A*	*D*—H⋯*A*
C12—H12⋯*Cg*1^i^	0.94	2.82	3.472 (6)	127
C22—H22⋯*Cg*1^ii^	0.94	2.72	3.565 (5)	149
C6—H6⋯*Cg*2^ii^	0.94	2.97	3.615 (7)	127

Symmetry codes: (i) 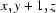; (ii) 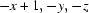.

**Fig. 1.**
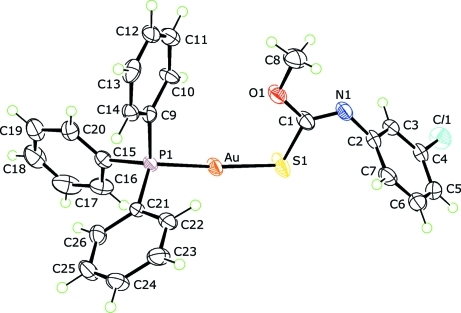
Molecular structure of (I) showing atom-labelling scheme and displacement ellipsoids at the 50% probability level.

**Fig. 2.**
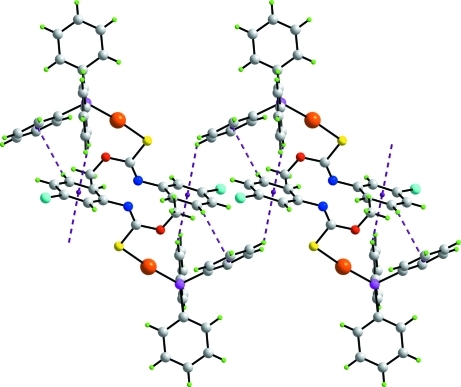
A supramolecular chain along the b axis, highlighting the pivotal role played by the N-bound benzene ring in the formation of donor and acceptor C–H···π interactions

**Fig. 3.**
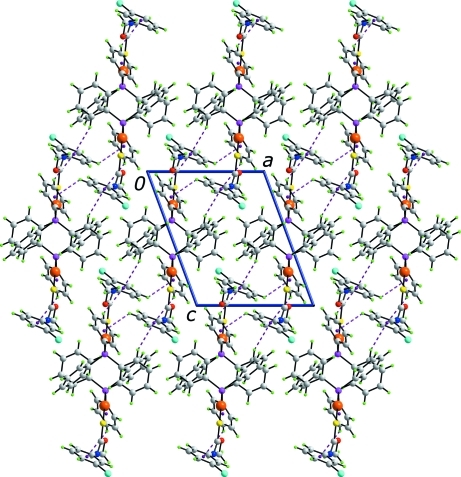
A view in projection along the b axis of the crystal packing in (I), highlighting the stacking of layers mediated by the C–H···π contacts (shown as purple dashed lines).

## supplementary crystallographic information

### Comment

The remarkable propensity of molecules with the general formula R_3_PAu[SC(OR')═ NR''], for R, R' and R'' = alkyl and aryl, to provide diffraction quality crystals have proved useful for systematic crystal engineering studies (Ho *et al.*, 2006; Ho & Tiekink, 2007; Kuan *et al.*, 2008). The structure of the title compound, (I), was investigated in the context of the above.

The nearly linear *SP* coordination geometry observed for the Au atom in (I), Fig. 1, is defined by phosphine and thiolate ligands, Table 1. The deviation from the ideal linearity [S—Au—P = 174.61 (4) °] is related to a short intramolecular Au···O contact [2.988 (3) Å]. With the exception of the *p*-tolyl derivatives (Kuan *et al.*, 2008), the overall molecular conformation (including close Au···.O contacts) conforms to the majority of related compounds having monodentate phosphine ligands.

The major feature of the crystal packing is the presence of C–H···π interactions that lead to the formation of supramolecular chains along the *b* axis, Fig. 2 and Table 2. Notably, the Cl-substituted benzene ring accepts two such interactions also donates one. Chains are arranged into layers in the (1 0 1) plane, Fig. 3.

### Experimental

Compound (I) was prepared following the standard literature procedure from the reaction of Ph_3_AuCl and MeOC(═S)N(H)(C_6_H_4_Cl-3) in the presence of NaOH (Hall *et al.*, 1993). Crystals were obtained by the slow evaporation of a CHCl_3_/hexane (3/1) solution held at room temperature.

### Refinement

The H atoms were geometrically placed (C—H = 0.94–0.97 Å) and refined as riding with *U*_iso_(H) = 1.2-1.5*U*_eq_(C). The maximum and minimum residual electron density peaks of 1.16 and 1.21 e Å^-3^, respectively, were located 0.84 Å and 0.88 Å from the Au atom.

### Crystal data

**Table:**

[Au(C_8_H_7_ClNOS)(C_18_H_15_P)]	*Z* = 2
*M**_r_* = 659.89	*F*(000) = 640
Triclinic, *P*1	*D*_x_ = 1.805 Mg m^−^^3^
Hall symbol: -P 1	Mo *K*α radiation, λ = 0.71069 Å
*a* = 10.4236 (8) Å	Cell parameters from 6911 reflections
*b* = 10.6961 (8) Å	θ = 1.8–30.1°
*c* = 12.7439 (9) Å	µ = 6.34 mm^−^^1^
α = 72.724 (2)°	*T* = 223 K
β = 66.105 (1)°	Block, colourless
γ = 72.530 (2)°	0.19 × 0.08 × 0.05 mm
*V* = 1213.83 (16) Å^3^	

### Data collection

**Table:**

Bruker SMART CCD diffractometer	5525 independent reflections
Radiation source: fine-focus sealed tube	4787 reflections with *I* > 2σ(*I*)
graphite	*R*_int_ = 0.023
ω scans	θ_max_ = 27.5°, θ_min_ = 1.8°
Absorption correction: multi-scan (*SADABS*; Bruker, 2000)	*h* = −11→13
*T*_min_ = 0.515, *T*_max_ = 1	*k* = −13→13
8630 measured reflections	*l* = −15→16

### Refinement

**Table:**

Refinement on *F*^2^	Primary atom site location: structure-invariant direct methods
Least-squares matrix: full	Secondary atom site location: difference Fourier map
*R*[*F*^2^ > 2σ(*F*^2^)] = 0.032	Hydrogen site location: inferred from neighbouring sites
*wR*(*F*^2^) = 0.089	H-atom parameters constrained
*S* = 1.05	*w* = 1/[σ^2^(*F*_o_^2^) + (0.0473*P*)^2^] where *P* = (*F*_o_^2^ + 2*F*_c_^2^)/3
5525 reflections	(Δ/σ)_max_ < 0.001
290 parameters	Δρ_max_ = 1.16 e Å^−^^3^
0 restraints	Δρ_min_ = −1.21 e Å^−^^3^

### Special details

**Table:**

Geometry. All s.u.'s (except the s.u. in the dihedral angle between two l.s. planes) are estimated using the full covariance matrix. The cell s.u.'s are taken into account individually in the estimation of s.u.'s in distances, angles and torsion angles; correlations between s.u.'s in cell parameters are only used when they are defined by crystal symmetry. An approximate (isotropic) treatment of cell s.u.'s is used for estimating s.u.'s involving l.s. planes.
Refinement. Refinement of *F*^2^ against ALL reflections. The weighted *R*-factor wR and goodness of fit *S* are based on *F*^2^, conventional *R*-factors *R* are based on *F*, with *F* set to zero for negative *F*^2^. The threshold expression of *F*^2^ > 2σ(*F*^2^) is used only for calculating *R*-factors(gt) etc. and is not relevant to the choice of reflections for refinement. *R*-factors based on *F*^2^ are statistically about twice as large as those based on *F*, and *R*- factors based on ALL data will be even larger.

### Fractional atomic coordinates and isotropic or equivalent isotropic displacement parameters (Å^2^)

**Table:**

	*x*	*y*	*z*	*U*_iso_*/*U*_eq_	
Au	0.11162 (2)	0.105503 (16)	0.249566 (14)	0.03318 (8)	
Cl1	0.29679 (17)	−0.34461 (14)	−0.25655 (13)	0.0480 (3)	
S1	0.1802 (2)	−0.03071 (14)	0.11917 (12)	0.0569 (5)	
P1	0.05527 (13)	0.25261 (11)	0.36314 (10)	0.0234 (2)	
O1	0.2184 (4)	0.2044 (3)	−0.0097 (3)	0.0418 (9)	
N1	0.3288 (5)	0.0379 (4)	−0.1105 (3)	0.0349 (9)	
C1	0.2538 (6)	0.0735 (5)	−0.0159 (4)	0.0341 (11)	
C2	0.3735 (5)	−0.0998 (5)	−0.1156 (4)	0.0316 (10)	
C3	0.3166 (5)	−0.1501 (5)	−0.1734 (4)	0.0314 (10)	
H3	0.2451	−0.0950	−0.2035	0.038*	
C4	0.3677 (5)	−0.2828 (5)	−0.1855 (4)	0.0317 (10)	
C5	0.4734 (6)	−0.3662 (5)	−0.1412 (4)	0.0371 (12)	
H5	0.5049	−0.4566	−0.1474	0.045*	
C6	0.5304 (6)	−0.3132 (6)	−0.0882 (5)	0.0409 (12)	
H6	0.6033	−0.3676	−0.0594	0.049*	
C7	0.4820 (6)	−0.1809 (5)	−0.0766 (4)	0.0363 (11)	
H7	0.5239	−0.1460	−0.0416	0.044*	
C8	0.2819 (8)	0.2932 (5)	−0.1111 (5)	0.0521 (15)	
H8A	0.3855	0.2663	−0.1339	0.078*	
H8B	0.2515	0.3829	−0.0956	0.078*	
H8C	0.2528	0.2917	−0.1739	0.078*	
C9	0.0939 (5)	0.4105 (4)	0.2678 (4)	0.0234 (9)	
C10	0.0614 (5)	0.4495 (5)	0.1642 (4)	0.0304 (10)	
H10	0.0210	0.3945	0.1462	0.037*	
C11	0.0890 (6)	0.5678 (5)	0.0901 (5)	0.0399 (12)	
H11	0.0651	0.5949	0.0219	0.048*	
C12	0.1513 (6)	0.6478 (5)	0.1140 (4)	0.0343 (11)	
H12	0.1703	0.7286	0.0618	0.041*	
C13	0.1852 (5)	0.6108 (5)	0.2118 (5)	0.0371 (12)	
H13	0.2286	0.6656	0.2271	0.044*	
C14	0.1562 (5)	0.4911 (4)	0.2909 (4)	0.0282 (10)	
H14	0.1791	0.4661	0.3594	0.034*	
C15	−0.1311 (5)	0.2901 (5)	0.4571 (4)	0.0275 (10)	
C16	−0.2001 (6)	0.1849 (6)	0.5219 (5)	0.0405 (12)	
H16	−0.1521	0.0965	0.5143	0.049*	
C17	−0.3406 (6)	0.2107 (7)	0.5980 (5)	0.0540 (17)	
H17	−0.3868	0.1393	0.6437	0.065*	
C18	−0.4121 (6)	0.3393 (7)	0.6068 (5)	0.0527 (16)	
H18	−0.5081	0.3558	0.6569	0.063*	
C19	−0.3451 (6)	0.4442 (6)	0.5434 (5)	0.0435 (13)	
H19	−0.3942	0.5322	0.5512	0.052*	
C20	−0.2039 (5)	0.4195 (5)	0.4673 (4)	0.0334 (11)	
H20	−0.1580	0.4913	0.4227	0.040*	
C21	0.1557 (5)	0.2115 (4)	0.4603 (4)	0.0239 (9)	
C22	0.3060 (5)	0.1834 (5)	0.4125 (4)	0.0351 (11)	
H22	0.3522	0.1829	0.3321	0.042*	
C23	0.3858 (6)	0.1567 (5)	0.4825 (5)	0.0407 (12)	
H23	0.4864	0.1408	0.4497	0.049*	
C24	0.3169 (6)	0.1532 (5)	0.6035 (5)	0.0387 (12)	
H24	0.3716	0.1345	0.6517	0.046*	
C25	0.1711 (6)	0.1769 (6)	0.6509 (4)	0.0398 (12)	
H25	0.1257	0.1725	0.7320	0.048*	
C26	0.0902 (5)	0.2070 (5)	0.5816 (4)	0.0340 (11)	
H26	−0.0104	0.2250	0.6153	0.041*	

### Atomic displacement parameters (Å^2^)

**Table:**

	*U*^11^	*U*^22^	*U*^33^	*U*^12^	*U*^13^	*U*^23^
Au	0.05238 (14)	0.02569 (11)	0.02024 (10)	−0.01321 (8)	−0.00694 (8)	−0.00573 (7)
Cl1	0.0597 (9)	0.0487 (8)	0.0462 (8)	−0.0174 (7)	−0.0213 (7)	−0.0137 (6)
S1	0.1055 (13)	0.0342 (7)	0.0234 (6)	−0.0339 (8)	0.0019 (7)	−0.0102 (5)
P1	0.0305 (6)	0.0229 (5)	0.0167 (5)	−0.0074 (5)	−0.0073 (4)	−0.0035 (4)
O1	0.069 (3)	0.0284 (17)	0.0234 (17)	−0.0170 (17)	−0.0080 (17)	−0.0037 (14)
N1	0.044 (2)	0.037 (2)	0.021 (2)	−0.0117 (19)	−0.0066 (18)	−0.0065 (17)
C1	0.050 (3)	0.033 (2)	0.023 (2)	−0.018 (2)	−0.009 (2)	−0.0052 (19)
C2	0.034 (3)	0.039 (3)	0.019 (2)	−0.009 (2)	−0.0034 (19)	−0.0090 (19)
C3	0.034 (3)	0.034 (2)	0.022 (2)	−0.005 (2)	−0.0081 (19)	−0.0022 (19)
C4	0.033 (3)	0.042 (3)	0.020 (2)	−0.012 (2)	−0.0041 (19)	−0.010 (2)
C5	0.036 (3)	0.034 (3)	0.031 (3)	−0.002 (2)	−0.006 (2)	−0.007 (2)
C6	0.033 (3)	0.052 (3)	0.030 (3)	−0.001 (2)	−0.010 (2)	−0.006 (2)
C7	0.039 (3)	0.047 (3)	0.024 (2)	−0.009 (2)	−0.013 (2)	−0.007 (2)
C8	0.074 (4)	0.036 (3)	0.039 (3)	−0.017 (3)	−0.015 (3)	0.002 (2)
C9	0.022 (2)	0.023 (2)	0.023 (2)	−0.0053 (17)	−0.0038 (17)	−0.0068 (17)
C10	0.047 (3)	0.030 (2)	0.021 (2)	−0.016 (2)	−0.018 (2)	0.0012 (18)
C11	0.054 (3)	0.038 (3)	0.027 (3)	−0.009 (2)	−0.017 (2)	−0.002 (2)
C12	0.047 (3)	0.025 (2)	0.022 (2)	−0.012 (2)	−0.003 (2)	−0.0001 (18)
C13	0.032 (3)	0.029 (2)	0.047 (3)	−0.010 (2)	−0.003 (2)	−0.015 (2)
C14	0.039 (3)	0.028 (2)	0.023 (2)	−0.007 (2)	−0.014 (2)	−0.0072 (18)
C15	0.029 (2)	0.035 (2)	0.025 (2)	−0.0115 (19)	−0.0122 (19)	−0.0060 (19)
C16	0.041 (3)	0.042 (3)	0.038 (3)	−0.017 (2)	−0.018 (2)	0.008 (2)
C17	0.039 (3)	0.072 (4)	0.047 (3)	−0.034 (3)	−0.017 (3)	0.021 (3)
C18	0.026 (3)	0.087 (5)	0.036 (3)	−0.008 (3)	−0.005 (2)	−0.010 (3)
C19	0.036 (3)	0.062 (4)	0.034 (3)	−0.006 (3)	−0.011 (2)	−0.017 (3)
C20	0.030 (3)	0.041 (3)	0.032 (3)	−0.008 (2)	−0.011 (2)	−0.011 (2)
C21	0.030 (2)	0.022 (2)	0.018 (2)	−0.0034 (17)	−0.0083 (17)	−0.0027 (16)
C22	0.034 (3)	0.039 (3)	0.022 (2)	−0.004 (2)	−0.007 (2)	0.000 (2)
C23	0.030 (3)	0.044 (3)	0.036 (3)	−0.002 (2)	−0.012 (2)	0.004 (2)
C24	0.038 (3)	0.048 (3)	0.031 (3)	−0.008 (2)	−0.019 (2)	0.001 (2)
C25	0.039 (3)	0.058 (3)	0.021 (2)	−0.009 (3)	−0.010 (2)	−0.007 (2)
C26	0.026 (2)	0.042 (3)	0.028 (2)	−0.003 (2)	−0.004 (2)	−0.010 (2)

### Geometric parameters (Å, °)

**Table:**

Au—P1	2.2416 (11)	C11—H11	0.9400
Au—S1	2.2902 (13)	C12—C13	1.348 (8)
Cl1—C4	1.731 (5)	C12—H12	0.9400
S1—C1	1.760 (5)	C13—C14	1.402 (6)
P1—C21	1.809 (5)	C13—H13	0.9400
P1—C9	1.813 (4)	C14—H14	0.9400
P1—C15	1.817 (5)	C15—C20	1.380 (7)
O1—C1	1.355 (6)	C15—C16	1.385 (6)
O1—C8	1.401 (6)	C16—C17	1.388 (8)
N1—C1	1.241 (6)	C16—H16	0.9400
N1—C2	1.418 (6)	C17—C18	1.367 (9)
C2—C7	1.374 (7)	C17—H17	0.9400
C2—C3	1.402 (7)	C18—C19	1.369 (8)
C3—C4	1.390 (7)	C18—H18	0.9400
C3—H3	0.9400	C19—C20	1.393 (7)
C4—C5	1.394 (7)	C19—H19	0.9400
C5—C6	1.372 (8)	C20—H20	0.9400
C5—H5	0.9400	C21—C22	1.404 (7)
C6—C7	1.384 (7)	C21—C26	1.406 (6)
C6—H6	0.9400	C22—C23	1.368 (8)
C7—H7	0.9400	C22—H22	0.9400
C8—H8A	0.9700	C23—C24	1.407 (7)
C8—H8B	0.9700	C23—H23	0.9400
C8—H8C	0.9700	C24—C25	1.362 (7)
C9—C14	1.375 (6)	C24—H24	0.9400
C9—C10	1.408 (6)	C25—C26	1.366 (7)
C10—C11	1.365 (7)	C25—H25	0.9400
C10—H10	0.9400	C26—H26	0.9400
C11—C12	1.376 (7)		
			
P1—Au—S1	174.61 (4)	C13—C12—C11	120.3 (5)
C1—S1—Au	102.46 (16)	C13—C12—H12	119.8
C21—P1—C9	105.6 (2)	C11—C12—H12	119.8
C21—P1—C15	104.4 (2)	C12—C13—C14	120.4 (5)
C9—P1—C15	105.7 (2)	C12—C13—H13	119.8
C21—P1—Au	115.81 (14)	C14—C13—H13	119.8
C9—P1—Au	107.75 (14)	C9—C14—C13	119.7 (4)
C15—P1—Au	116.62 (15)	C9—C14—H14	120.1
C1—O1—C8	116.8 (4)	C13—C14—H14	120.1
C1—N1—C2	120.4 (4)	C20—C15—C16	119.6 (5)
N1—C1—O1	120.4 (4)	C20—C15—P1	121.9 (4)
N1—C1—S1	126.6 (4)	C16—C15—P1	118.6 (4)
O1—C1—S1	113.0 (3)	C15—C16—C17	119.6 (5)
C7—C2—C3	119.4 (5)	C15—C16—H16	120.2
C7—C2—N1	121.4 (5)	C17—C16—H16	120.2
C3—C2—N1	118.8 (5)	C18—C17—C16	120.4 (5)
C4—C3—C2	118.9 (5)	C18—C17—H17	119.8
C4—C3—H3	120.6	C16—C17—H17	119.8
C2—C3—H3	120.6	C17—C18—C19	120.6 (5)
C3—C4—C5	121.4 (5)	C17—C18—H18	119.7
C3—C4—Cl1	118.7 (4)	C19—C18—H18	119.7
C5—C4—Cl1	119.9 (4)	C18—C19—C20	119.6 (5)
C6—C5—C4	118.5 (5)	C18—C19—H19	120.2
C6—C5—H5	120.8	C20—C19—H19	120.2
C4—C5—H5	120.8	C15—C20—C19	120.3 (5)
C5—C6—C7	120.9 (5)	C15—C20—H20	119.9
C5—C6—H6	119.6	C19—C20—H20	119.9
C7—C6—H6	119.6	C22—C21—C26	118.3 (4)
C2—C7—C6	120.9 (5)	C22—C21—P1	118.6 (3)
C2—C7—H7	119.6	C26—C21—P1	123.1 (4)
C6—C7—H7	119.6	C23—C22—C21	120.3 (5)
O1—C8—H8A	109.5	C23—C22—H22	119.8
O1—C8—H8B	109.5	C21—C22—H22	119.8
H8A—C8—H8B	109.5	C22—C23—C24	119.9 (5)
O1—C8—H8C	109.5	C22—C23—H23	120.0
H8A—C8—H8C	109.5	C24—C23—H23	120.0
H8B—C8—H8C	109.5	C25—C24—C23	120.0 (5)
C14—C9—C10	119.2 (4)	C25—C24—H24	120.0
C14—C9—P1	123.0 (4)	C23—C24—H24	120.0
C10—C9—P1	117.8 (3)	C24—C25—C26	120.7 (5)
C11—C10—C9	119.5 (4)	C24—C25—H25	119.7
C11—C10—H10	120.2	C26—C25—H25	119.7
C9—C10—H10	120.2	C25—C26—C21	120.7 (5)
C10—C11—C12	120.8 (5)	C25—C26—H26	119.6
C10—C11—H11	119.6	C21—C26—H26	119.6
C12—C11—H11	119.6		
			
P1—Au—S1—C1	−8.4 (7)	C11—C12—C13—C14	−0.6 (8)
S1—Au—P1—C21	−104.7 (7)	C10—C9—C14—C13	0.5 (7)
S1—Au—P1—C9	13.3 (7)	P1—C9—C14—C13	178.8 (3)
S1—Au—P1—C15	131.8 (7)	C12—C13—C14—C9	0.6 (7)
C2—N1—C1—O1	175.9 (5)	C21—P1—C15—C20	95.9 (4)
C2—N1—C1—S1	−5.4 (8)	C9—P1—C15—C20	−15.2 (5)
C8—O1—C1—N1	−6.2 (8)	Au—P1—C15—C20	−134.9 (4)
C8—O1—C1—S1	175.0 (4)	C21—P1—C15—C16	−82.6 (4)
Au—S1—C1—N1	166.0 (5)	C9—P1—C15—C16	166.2 (4)
Au—S1—C1—O1	−15.3 (4)	Au—P1—C15—C16	46.6 (4)
C1—N1—C2—C7	−75.8 (7)	C20—C15—C16—C17	−1.3 (8)
C1—N1—C2—C3	111.4 (6)	P1—C15—C16—C17	177.3 (4)
C7—C2—C3—C4	2.6 (7)	C15—C16—C17—C18	1.8 (9)
N1—C2—C3—C4	175.6 (4)	C16—C17—C18—C19	−1.9 (10)
C2—C3—C4—C5	0.2 (7)	C17—C18—C19—C20	1.5 (9)
C2—C3—C4—Cl1	179.8 (3)	C16—C15—C20—C19	0.9 (8)
C3—C4—C5—C6	−2.2 (7)	P1—C15—C20—C19	−177.6 (4)
Cl1—C4—C5—C6	178.2 (4)	C18—C19—C20—C15	−1.0 (8)
C4—C5—C6—C7	1.4 (8)	C9—P1—C21—C22	−65.8 (4)
C3—C2—C7—C6	−3.4 (7)	C15—P1—C21—C22	−177.0 (4)
N1—C2—C7—C6	−176.2 (5)	Au—P1—C21—C22	53.3 (4)
C5—C6—C7—C2	1.4 (8)	C9—P1—C21—C26	113.8 (4)
C21—P1—C9—C14	−14.8 (4)	C15—P1—C21—C26	2.6 (4)
C15—P1—C9—C14	95.5 (4)	Au—P1—C21—C26	−127.1 (4)
Au—P1—C9—C14	−139.1 (3)	C26—C21—C22—C23	−2.0 (7)
C21—P1—C9—C10	163.6 (4)	P1—C21—C22—C23	177.6 (4)
C15—P1—C9—C10	−86.1 (4)	C21—C22—C23—C24	2.0 (8)
Au—P1—C9—C10	39.2 (4)	C22—C23—C24—C25	−0.3 (9)
C14—C9—C10—C11	−1.5 (7)	C23—C24—C25—C26	−1.4 (9)
P1—C9—C10—C11	−180.0 (4)	C24—C25—C26—C21	1.4 (8)
C9—C10—C11—C12	1.6 (8)	C22—C21—C26—C25	0.4 (7)
C10—C11—C12—C13	−0.5 (8)	P1—C21—C26—C25	−179.2 (4)

### Hydrogen-bond geometry (Å, °)

**Table:**

Cg1 and Cg2 are the centroids of the C2–C7 and C9–C14 rings, respectively.

**Table:**

*D*—H···*A*	*D*—H	H···*A*	*D*···*A*	*D*—H···*A*
C12—H12···Cg1^i^	0.94	2.82	3.472 (6)	127
C22—H22···Cg1^ii^	0.94	2.72	3.565 (5)	149
C6—H6···Cg2^ii^	0.94	2.97	3.615 (7)	127

Symmetry codes: (i) *x*, *y*+1, *z*; (ii) −*x*+1, −*y*, −*z*.
